# Physcion Protects Against Ethanol-Induced Liver Injury by Reprogramming of Circadian Clock

**DOI:** 10.3389/fphar.2020.573074

**Published:** 2020-11-23

**Authors:** Youli Yao, Along Zuo, Qiyu Deng, Shikang Liu, Tianying Zhan, Maolin Wang, Haidong Xu, Junxian Ma, Yingying Zhao

**Affiliations:** ^1^ Department of Physiology, School of Basic Medical Sciences, Shenzhen University Health Sciences Center, Shenzhen University, Shenzhen, China; ^2^ Key Laboratory for Natural Resource of Changbai Mountain and Functional Molecules, Ministry of Education, Yanbian University, Yanji, China; ^3^ University of Chinese Academy of Sciences, Shenzhen Hospital, Shenzhen, China; ^4^ School of Information Engineering, Shenzhen University, Shenzhen, China

**Keywords:** alcoholic liver steatosis, physcion, circadian clock, lipogenesis, inflammation

## Abstract

The circadian clock plays a key role in our daily physiology and metabolism. Alcohol consumption disrupts the circadian rhythm of metabolic genes in the liver; however, the potential contribution of circadian clock modulation to alcoholic liver disease (ALD) is unknown. We identified a novel liver protective agent, physcion, which can alleviate fat accumulation and inflammation in ALD mice via reprogramming the hepatic circadian clock. The model of alcoholic hepatitis was established by intragastrically administering ethanol. *In vitro*, physcion was investigated by treating HepG2 cells with ethanol. The role of circadian clock in Physcion caused liver protection was tested by knocking down the core circadian gene Bmal1. Physcion application caused reduced lipogenesis and alleviated inflammation in alcohol-induced mice. In alcoholic hepatosteatosis models, physcion upregulated the core circadian genes. And the circadian misalignment triggered by ethanol was efficiently reversed by physcion. Physcion attenuated lipogenesis *via* reprogramming the circadian clock in HepG2 cells. Suppression of Bmal1 by RNA interference abolished the protective of physcion. In addition, Physcion binds to the active pocket of BMAL1 and promotes its expression. The study identified the novel liver protective effects of physcion on alcohol-induced liver injury, and modulation of the core circadian clock regulators contributes to ALD alleviation. More importantly, strategies targeting the circadian machinery, for example, Bmal1, may prove to be beneficial treatment options for this condition.

## Introduction

Alcoholic liver disease (ALD) originates from excessive alcohol abuse and includes simple liver steatosis, steatohepatitis, fibrosis and/or cirrhosis (Gustot and Jalan, 2019). The etiopathogenesis of ALD is complex. ALD is caused by the interactivity between genetic factors, environmental factors and metabolic-related processes following heavy alcohol consumption (Mukherji et al., 2019). Alcohol usage imbalances the metabolic processes of lipids and increasing the amount of triglycerides in liver cells (Wang et al., 2016). This precipitates injury to liver cells. The disruption of lipid metabolism in liver cells marks the initiation of ALD. Disruption of β-oxidation in convert with high fatty acid synthesis increases lipid accumulation. Alcohol consumption directly or indirectly regulates sterol regulatory element binding protein 1 (SREBP-1) and peroxisome proliferator-activated receptor-a (PPARα), promotes lipogenesis and inhibits the oxidation of fatty acids. Adenosine monophosphate-activated protein kinase (AMPK) is a metabolic sensor that can inhibit lipid synthesis and accelerate fatty acid oxidation by regulating the activity of SREBP-1 and PPARα.

It has been reported that circadian rhythm disorders may be the basis of alcohol-induced tissue damage (Udoh et al., 2015). Mice with a disturbed circadian rhythm have been reported to have higher intestinal epithelial barrier permeability, which is a risk factor for alcohol tissue damage (Summa et al., 2013). Shift workers exhibit increased alcohol intake and a tendency to engage in binge drinking (Virtanen et al., 2015). The circadian rhythm is an endogenous biological process that relies on the molecular clock. The central clock driving the rhythmic behavior is located in the suprachiasmatic nucleus in the anterior hypothalamus, and the sub-clock is located in other peripheral tissue cells (e.g., liver, kidney, heart) (Chang and Guarente, 2013). The central clock serves to regulate and coordinate the surrounding organization’s biological clock to keep pace with the central clock (Zhang S. L. et al., 2018).

The brain and muscle arnt-like protein-1 (Bmal1) gene is the only clock gene whose deletion completely disrupts all biological rhythms (Early et al., 2018). In hepatocytes, activation of Bmal1 involves cyclic expression of various metabolic genes involved in lipid, glucose and cholesterol metabolism (Asher and Sassone-Corsi, 2015). A lack of liver Bmal1 leads to severe insulin resistance and hepatic steatosis after feeding on a chronic high-fat diet (Jacobi et al., 2015). Liver-specific knockout or depletion of Bmal1 in ethanol-fed mice leads to more severe hepatic steatosis and damage (Zhang D. et al., 2018). In addition, the compensatory growth of β-cells caused by diet-induced obesity-related insulin increases is controlled by Bmal1 (Rakshit et al., 2016). These observations suggest that the core circadian gene Bmal1 is involved in fatty acid synthesis and the β-oxidation pathway (Mi et al., 2017b). Therefore, regulating circadian rhythm disorders caused by alcohol feeding may be a treatment target for ALD. The studies have discovered that EGCG and tea polyphenols reduce insulin resistance in a Bmal1-dependent way and by activating AMPK signaling pathways (Mi et al., 2017a; Mi et al., 2017b). Besides, proanthocyanidins can regulate circadian gene expression in cultured fibroblasts (Ribas-Latre et al., 2015). However, there are hitherto not many pharmacological agents available to protect the liver by adjusting its peripheral circadian rhythm.

*Rheum emodi* is a source of important foods in a variety of different forms and used as a cooking plant around the world (Arvindekar et al., 2015). The rhizomes are usually cooked after drying, produce sour when stewed, and are used to make wine and baked goods (Clementi and Misiti, 2010). *Rheum emodi* has a wide range of uses in the chemical, pharmaceutical, cosmetic and food industries (Arvindekar et al., 2015).

**FIGURE 1:**
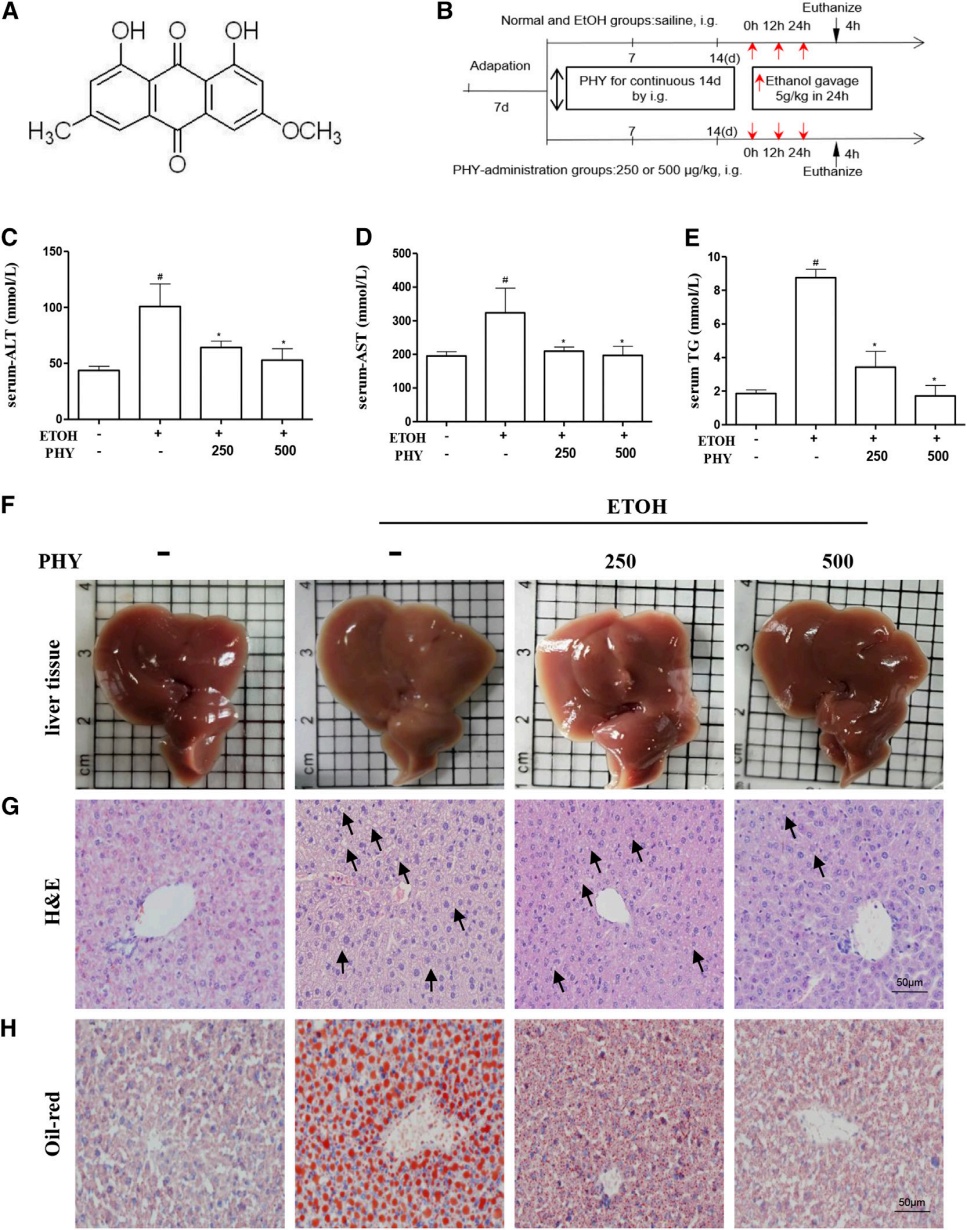
Physcion reduces the symptoms of ethanol-induced hepatic injury. **(A)** Chemical structure of physcion, **(B)** animal experimental procedure, **(C–E)** serum ALT, AST, and TG contents, **(F)** liver appearance pictures, **(G)** H&E staining, and **(H)** Oil Red O staining. Values significantly different from the control are indicated by hash signs (^#^ *p* < 0.05). Values significantly different from the ethanol are indicated by asterisks (*p < 0.05).

Physcion (1,8-dihydroxy-3-methoxy-6-methyloxime, Figure 1A), a natural anthraquinone derivative, is derived from *Rheum emodi* and *Cortinariuspurpurascens Fr* (Agarwal et al., 2000). Physcion is also present in cabbage lettuce, beans, and *Cassia obtusifolia* (Mueller et al., 1999). It has been reported to possess a variety of pharmacological properties, including anti-inflammatory, anti-oxidant, laxative and anti-microbial activities (Agarwal et al., 2000; Zhao et al., 2009). It was reported that the Physcion, when used as dietary supplement, improved diet-induced obesity and its complications (Lee et al., 2019). Little is known about the pharmacological effects of Physcion on circadian parameters, and the question whether and how Physcion protects the liver from acute alcoholic hepatosteatosis by regulation Bmal1 remained unclear. Our results reveal that Physcion can ameliorate alcoholic hepatosteatosis through the modulation of BMAL1 and AMPK/SREBP1/P2X7R signaling.

## Materials and Methods

### Materials

Physcion was purchased from MedChemExpress. Horse serum was purchased from GIBCO Company. Anti-SREBP-1, anti-PPARα, anti-CLOCK, and anti-BMAL1 antibodies were purchased from Abcam (Cambridge, MA, United States). Anti-AMPKα, anti-phospho-AMPKα, and anti-cleaved caspase-1 antibodies were purchased from Cell Signaling Technology (Beverly, MA, United States). Anti-P2X7R, anti-IL1β, and anti-caspase1 antibodies were purchased from Santa Cruz Biotechnology. The BCA Protein Assay Kit was purchased from Solarbio (Beijing, China).

### Animals

In this investigation, 8–10-week-old male C57BL/6 (25–28 g) mice were bought from the Guangdong Medical Laboratory Animal Center (Guangzhou, China) [(SPF, SCXK (Yue)2018-0002), Guangzhou, China]. They were allowed to stabilize to a temperature-a humidity-controlled room (23 ± 2°C) with a 12:12-h light-dark cycle throughout in the study and SPF. The relative humidity was 50–60%. All tests and protocols performed in this study were permitted by the Institutional Animal Care and Use Committee of Shenzhen University (Resolution no. 2019005).

The mice (250 or 500 μg kg^−1^ body weight) were randomly divided into a normal group, a model group, a low-concentration Physcion treatment group (250 μg kg^−1^ body weight), and a high-concentration Physcion treatment group (500 μg kg^−1^ body weight) (Figure 1B), with eight mice in each group (Figure 1B) (Portari et al., 2016; Yao et al., 2017). Doses of Physcion were determined according to previous publications (Shen et al., 2011; Moon et al., 2019). Physcion administration groups were pretreated with single dose of physcion by oral gavage for 14 consecutive days. At the same time, control group and ethanol group were administrated equal volumes of saline by gavage for 14 consecutive days. Next, the model group and the treatment group were given ethanol (5 g kg^−1^ body weight) every 12 h for a total of three doses. After 4 h, blood and liver tissue were collected. A part of the liver tissue was fixed in 10% formalin, and the rest was stored at −80°C for subsequent analysis.

### Cell Culture

HepG2 cells (human hepatoma cell line) were purchased from ATCC and cultured in DMEM at 37°C under 5% CO_2_. After trypsinization and passaging, cells were added into 6-well plates (5 × 10^5^ cells/well). The treatment group comprised cells of fourth to seventh passages which were treated with physcion (0.125, 0.25 μm) and EtOH (50 mm) for 24 h (Moon et al., 2019). For circadian synchronization by serum, cells were treated with medium containing 50% horse serum for 2 h and treated with EtOH (50 mM) or PHY (0.25 μM) for 24 h. The cells were prepared for qPCR to measure mRNA level at 4 h intervals from 24 h (ZT = 0) to 44 h (ZT = 24) time-points.

### Western Blot Assays

The proteins were extracted from tissues by homogenization in RIPA reagent (Sigma), and protein concentrations were determined by BCA Protein Assay Kit (Solarbio, Beijing, China). Proteins from each group were subjected to into analysis. The proteins were resolved by SDS/PAGE followed electrotransfer to PVDF membrane (Amersham Hybond, GE Healthcare Bio-Sciences, Pittsburgh, PA, United States). The PVDFs were incubated with the primary antibody in QuickBlock^™^ primary antibody dilution buffer (P0256, Beyotime, Haimen, China). The next day, remove the primary resistance, the primary antibody was detected with the secondary antibody in PBS containing 0.05% Tween 20, and then chemiluminescenced *via* ECL (RPN2232, GE Healthcare, United Kingdom). The protein expression was normalized to that of GAPDH as the internal loading using Quantity One software (RRID:SCR_014280, Bio-Rad, CA, United States).

### RNA Isolation and Quantitative Real-Time PCR Analysis

The MiniBEST Universal RNA Extraction Kit was used to extract total RNA from liver tissues according to manufacturer’s protocol (TaKaRa, Dalian, China). Further processing of the RNA involved on determination of RNA purity, after which it was transcribed to cDNA by the PrimeScript RT Master Mix kit. Real-time PCR performed on Power SYBR^®^ Green PCR Master Mix on an Analytik jena QPCR System was utilized quantify mRNA level. GAPDH served as a housekeeping gene. The threshold cycle (ΔΔCt) method was applied in mRNA level determination. The primers for PCR are shown in Table 1.

**TABLE 1:** Primer sequences used for quantitative RT-PCR analysis.

Human primer sequences used for quantitative real-time PCR analysis		
Genes	Forward (5′–3′)	Reverse(5′–3′)
hBmal1	5′-TATCAGGCCAGGCTCAGGAGAAC-3′	5′-CCAGTCCAGCATCTGCTTCCAAG-3′
hPer2	5′-CTACACCGTGGAGGAGATGGAGAG-3′	5′-ACTTGGCATCGCTGAAGGCATC-3′
hPer1	5′-CAGGCAACGGCAAGGACTCAG-3′	5′-GGAGGCTGTAGGCAATGGAACTG-3′
hClock	5′-GTGACTGCTCCTGTAGCTTGTGG-3′	5′-TGCTGCTGCTGCGTTACTGAC-3′
hCry1	5′-CACCATCCGCTGCGTCTACATC-3′	5′-ACATCTGCTGGTTGTCCACGAATC-3′
hCry2	5′-CTCTCCTGCCGCCTCTTCTACTAC-3′	5′-GGTTGTTGGTAGCTGCCGTGTAG-3′

Mouse primer sequences used for quantitative real-time PCR analysis		
	Forward	Reverse
mIL1β	5′- GTACATCAGCACCTCACAAG-3′	5′-CACAGGCTCTCTTTGAACAG-3′
mSrebp1	5′-CTTAGCCTCTACACCAACTG-3′	5′-AGGAATACCCTCCTCATAGC-3′
mPparα	5′-GTCACACAATGCAATTCGCTTT-3′	5′-TTTGCTTTTTCAGATCTTGGCA-3′
mP2x7r	5′-CTTGAGTCGGCAAAGAAATC-3′	5′-GAGATGGTCAATGGCAGAAC-3′
mcaspase1	5′-ACTCGGGTGGAAGAAGACAG-3′	5′-CTCCTCAGCAAATCGGAACTG-3′
mFasn	5′-ATTGTGGATGGAGGTATCAAC-3′	5′-CTGGTAGGCATTCTGTAGTG-3′
mBmal1	5′-ACAATGAGCCAGACAACG-3′	5′-TTCCCATCTATTGCGTGT-3′
mPer2	5′-CACTTGCCTCCGAAATAA-3′	5′-ACTACTGCCTCTGGACTGG-3′
mNlrp3	5′-GAGCTGGACCTCAGTGACAATGC-3′	5′-ACCAATGCGAGATCCTGACAACAC-3′
mGAPDH	5′-CTTGTGCAGTGCCAGCC-3′	5′-GCCCAATACGGCCAAATCC-3′

### Oil Red O Staining and H&E Staining Analysis

A section of the liver tissue was quickly frozen by submerging it in Tissue-Tek OCT compound. The frozen tissues were then cut on a microtome into 5 µm slices and blocked with Oil Red O solution or stained with H&E solution at room temperature. The tissue specimen was finally sealed using glycerin gelatin and histopathological sections were examined by Nikon TI-E fluorescence microscope (Nikon, Tokyo, Japan).

### Immunofluorescence Analysis

The frozen sections were fixed in acetone/methanol (1:1), dried, dehydrated. Immunofluorescence analysis of cells experiments were performed after washing the cells three times with PBS and fixing them in 10% paraformaldehyde and permeating them with 0.1% Triton X-100. Cryosections or cells were treated with a rabbit BMAL1 antibody overnight at 4°C. The Cryosections or cells were incubated with Alexa Fluor^®^ 488 goat anti-rabbit IgG (ab150077, Abcam, Cambridge, MA, United States) followed by counterstaining with DAPI. The fluorescence was visualized by a confocal microscope (LSM 510 Meta; Carl Zeiss, Oberkochen, Germany).

### Computer Analysis

For computer analysis, AutoDock version 4.2.6 was used to perform molecular docking and analyze the interaction between Physcion and Bmal1. The three-dimensional structure of Bmal1 was obtained from the RCSBPDB database (https://www.rcsb.org/). Before docking, the protein is protonated and the water molecules in the structure are deleted. The structure of Physcion is obtained from the Pubchem database (https://pubchem.ncbi.nlm.nih.gov/).

### Statistical Analyses

All data are showed as the mean ± SD and significant differences were defined as *p* < 0.05. Group comparisons carried out with one-way analysis of variance and Tukey’s multiple comparison tests as appropriate. All analyses were performed on GraphPad Prism.

## Results

### Physcion Reduces the Symptoms of Ethanol-Induced Hepatic Injury

A mouse model of binge drinking model was established by exposing mice to ethanol (5 g kg^−1^) for 24 h (Figure 1B). The acute ethanol challenge induced significant increases in serum ALT and AST levels (Figures 1C,D). An increase in serum TG levels was observed in mice that received ethanol compared to the control group (Figure 1E). The changes induced by ethanol intake were abolished by physcion pretreatment which decreased the concentration of ALT, TG, and AST in serum levels, dose-dependently. We further verified the effects of physcion on acute ethanol-induced hepatic injury by liver tissue imaging, H&E staining, and Oil Red O staining. As shown in Figure 1F, livers in the EtOH group presented greasy and were easily disrupted compared with livers in the control group; this was prevented by physcion treatment. Ethanol exposure resulted in massive fat vacuole accumulation compared with the control group. The fat vacuoles of physcion-pretreated mouse livers were much smaller than those in the EtOH group (Figure 1G). The obvious fat droplets were also exhibited in liver sections from mice given ethanol, by contrast, administration of physcion prevented ethanol-induced acute hepatic steatosis (Figure 1H).

### Physcion Effectively Reduce the Lipogenesis Induced by Ethanol

**FIGURE 2:**
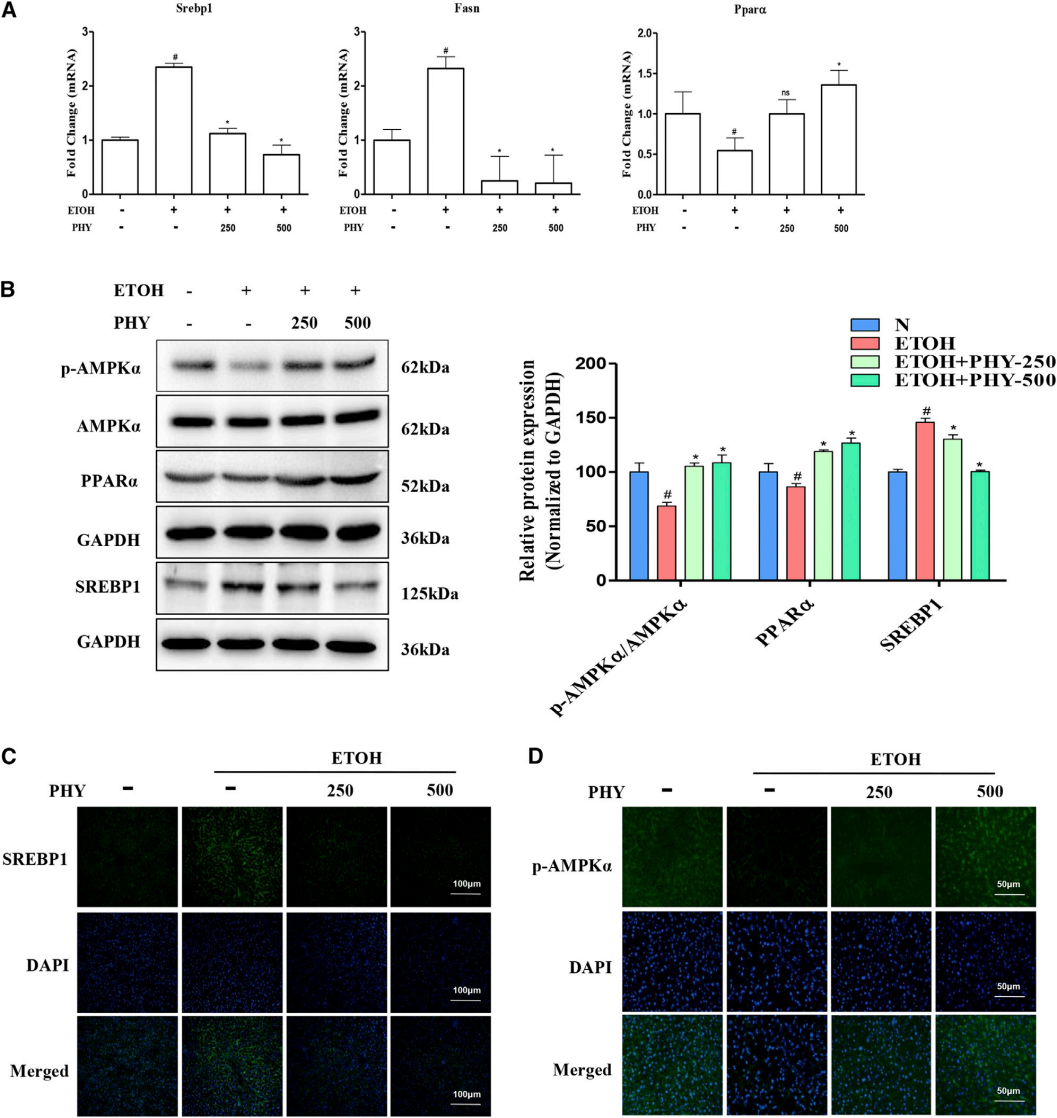
Physcion effectively reduce the lipogenesis induced by ethanol. **(A)** The mRNA expression of Srebp1, Fasn, and Pparα. **(B)** The protein expression of AMPKα, *p*-AMPKα, PPARα, and SREBP1. **(C)** Immunofluorescence staining of SREBP1 (×400 original magnification). **(D)** The phospho-AMPKα staining (green) and nuclei with DAPI (blue) are shown. Values significantly different from the control are indicated by hash signs (^#^ *p* < 0.05). Values significantly different from the ethanol are indicated by asterisks (*p < 0.05).

It has been reported that ethanol feeding significantly suppresses the AMPK-SREBP1 lipid metabolic pathway. Next, we studied the effect of Physcion on the AMPK pathway. Consistent with H&E results, the elevation of Srebp1 and Fasn mRNA levels was prevented by Physcion in acute alcohol-induced liver injury (Figure 2A). The Physcion-induced depress in hepatic SREBP1 protein expression was further confirmed by western blotting analysis (Figure 2B). The expression of PPARα and *p*-AMPKα were reduced by ethanol challenge and was restored by Physcion to nearly normal levels (Figure 2B).

To further detect the distribution of SREBP-1 and *p*-AMPKα in liver tissues, Immunofluorescence analysis was performed on histological sections of liver tissues. Positive staining of SREBP-1 was obviously observed in the liver section from EtOH group. And Physcion treatment decreased the positive staining of SREBP-1 compared with EtOH group (Figure 2C). The positive (in green) expressions of *p*-AMPKα in EtOH group were decreased, while these were inhibited by Physcion treatment (Figure 2D).

### Physcion Suppresses Inflammasome Activation in Ethanol-Induced Acute Hepatic Injury

**FIGURE 3:**
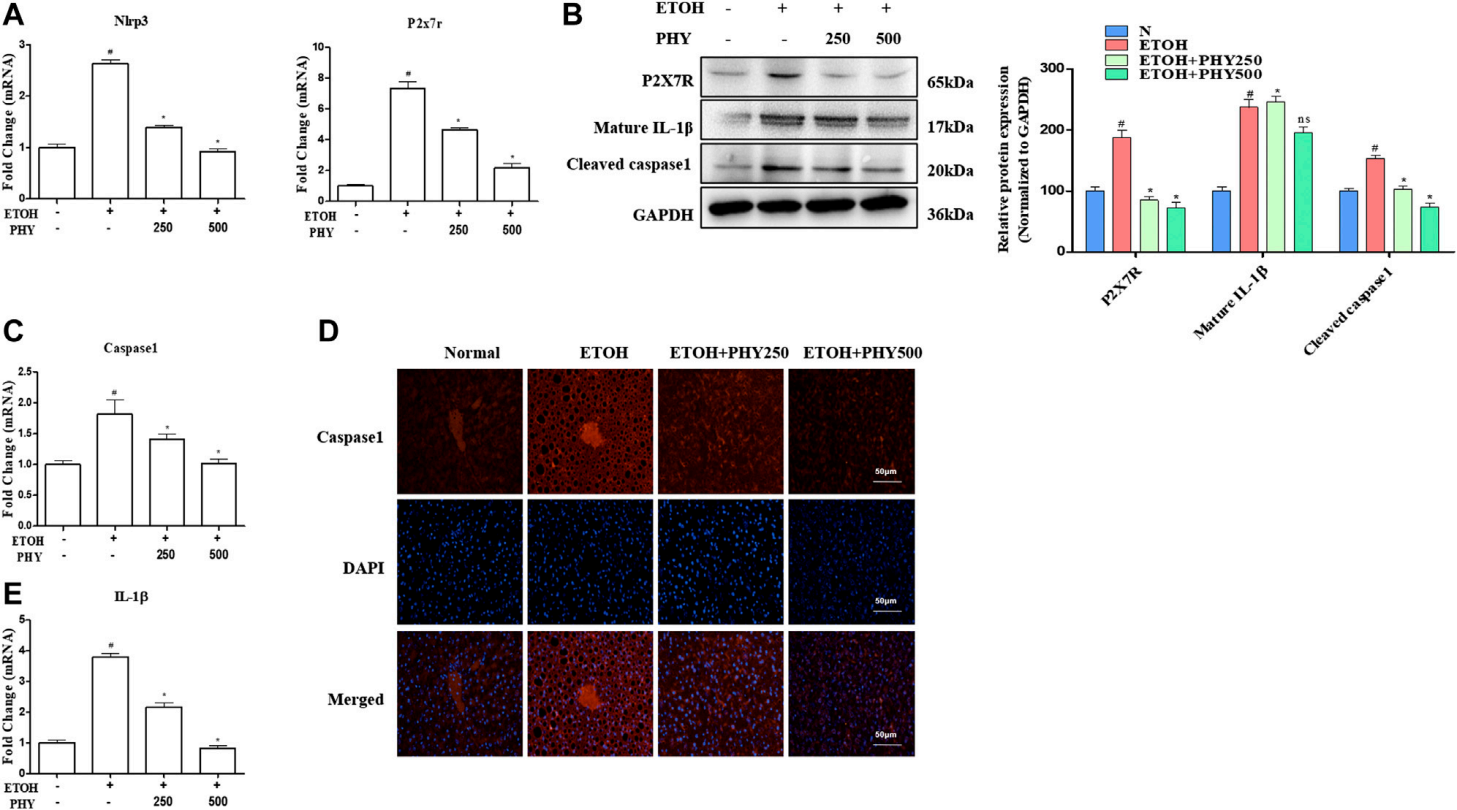
Physcion suppresses inflammasome activation in ethanol-induced acute hepatic injury. **(A)** The mRNA expression of Nlrp3 and P2x7r. **(B)** The protein expression levels of P2X7R, and IL-1β. **(C)** The mRNA expression of Caspase1. **(D)** Caspase-1 staining (red) and nuclei with DAPI (blue) are shown. **(E)** The mRNA expression of IL-1β. Values significantly different from the control are indicated by hash signs (^#^ *p* < 0.05). Values significantly different from the ethanol are indicated by asterisks (*p < 0.05).

Relatively low levels of NLRP3 and P2X7R were detected in the normal mouse liver. Alcohol administration increased NLRP3 and P2X7R expression in the mouse liver, as shown by western blotting or qPCR (Figures 3A,B). Physcion administration also significantly reduced NLRP3 and P2X7R expression (Figures 3A,B). In addition, we observed an apparent elevation in the mRNA levels of Caspase-1 in the ethanol-treated acute steatosis mouse liver compared with those in the liver of control mice (Figure 3C). A similar result was observed with the Caspase-1 staining assay in the mouse liver (Figure 3D). To investigate the involvement of NLRP3 inflammasome, the expression of the activation of caspase one and cleavage of IL-1 beta were detected by western blotting (Figure 3B). Mice treated with ethanol showed an obvious increase in the expression of the activation of caspase one and cleavage of IL-1β, demonstrating that P2X7 receptor-NLRP3/caspase-1 inflammasome activation contributes to an elevation in IL-1β production (Figure 3E). Pretreatment with Physcion significantly blocked IL-1β secretion and Caspase-1 activation (Figures 3B–E).

### Physcion Improves the Disorder of the Circadian Clock Pathway in Acute Alcoholic Hepatosteatosis

**FIGURE 4:**
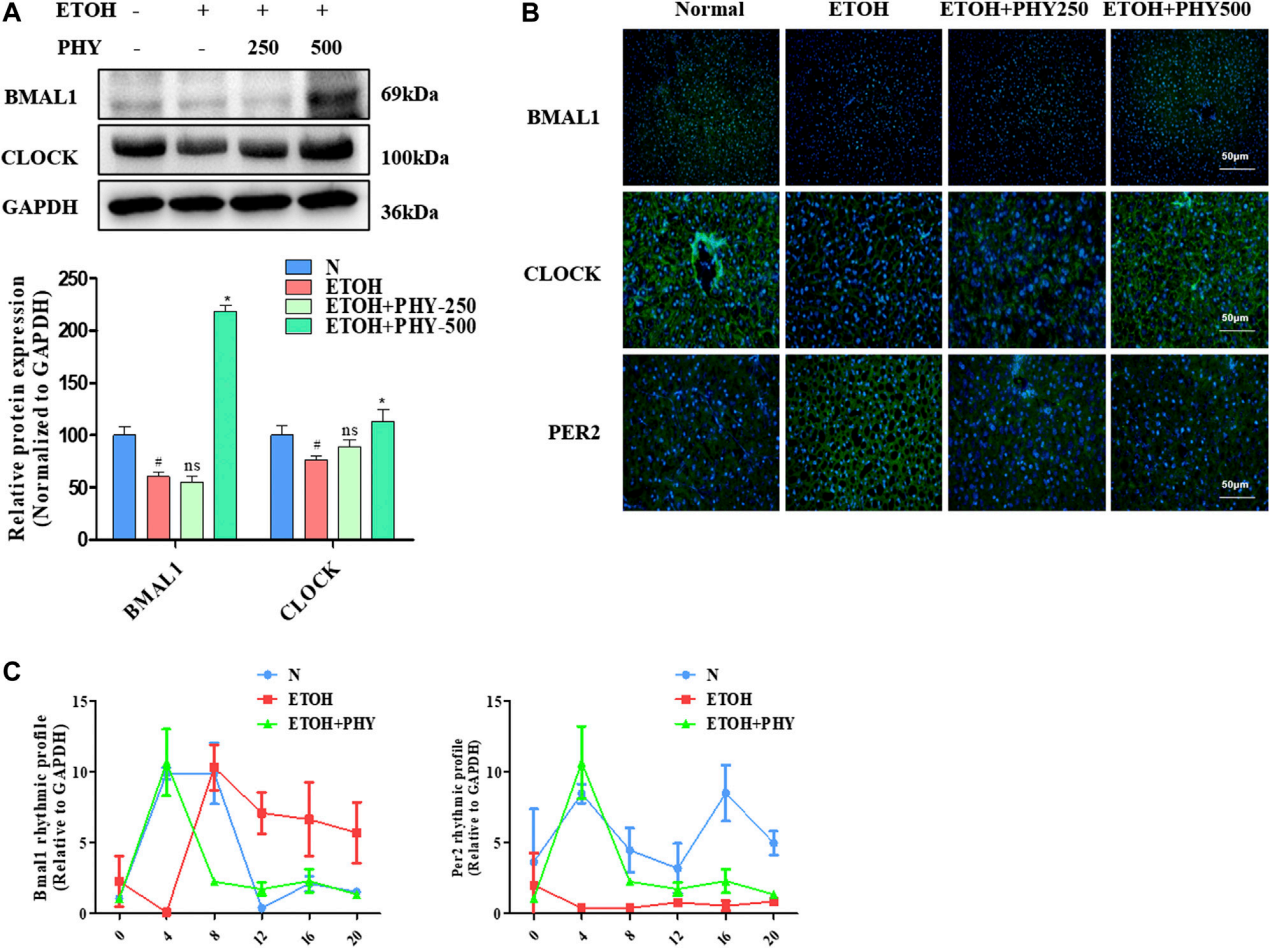
Physcion improves the disorder of the circadian clock pathway in acute alcoholic hepatosteatosis. **(A)** The protein expression of BMAL1, and CLOCK. **(B)** Immunofluorescence staining of BMAL1, CLOCK, and PER2. **(C)** The mRNA expression of Bmal1 and Per2. Values significantly different from the control are indicated by hash signs (^#^ *p* < 0.05). Values significantly different from the ethanol are indicated by asterisks (*p < 0.05).

The circadian clock in peripheral liver tissues regulates the rhythmic expression and activity of proteins with cellular and physiological functions, including lipid metabolic and inflammatory pathways. The core components of the circadian mechanism are transcriptional activators, CLOCK and BMAL1, as well as their targets, CRYs and PERs (Rutter et al., 2002). The protein expression levels of CLOCK and BMAL1 were reduced as a result of ethanol application, whereas physcion treatment could recover their expression in a dose-dependent manner, in some cases even reaching control values (Figure 4A).

We further evaluated the protein expression levels of CLOCK and BMAL1 by immunofluorescence, which were consistent with the results of Western blotting. The immunofluorescence analysis revealed a reduction in ethanol-treated groups that was inhibited in mice receiving ethanol and physcion (Figure 4B). Next, we measured changes in PER2 expression by immunofluorescence analysis. The immunofluorescence analysis revealed an increase in ethanol-treated groups that was significantly inhibited in the mice receiving ethanol and physcion (Figure 4B).

Studies have found that an alcohol diet impairs the circadian rhythm and metabolic cycles (Zhang D. et al., 2018). We designed the following experiments to reveal the underlying mechanism of the circadian clock to alleviate metabolic disorders, mice were given ethanol (5 g kg^−1^) three times in 24 h to induce metabolic disorders. Physcion administration mice were retreated with a dose of physcion by oral gavage for 14 successive days. Animals were euthanized and collected at 4 h intervals for mRNA analysis. The ability of physcion to regulate the molecular clock was demonstrated by monitor mRNA level of Bmal1 and Per2 for 24 h. The ethanol triggers the phase shift of the biological clock and the amplitude of oscillations of Per2 was significantly attenuated (Figure 4C). Interestingly, Physcion pretreatment leads to relative reversal daily shallow oscillation and phase shift of circadian clock.

### Physcion Improves Increased Inflammasome Activation and Ameliorates Lipid Accumulation in HepG2 Cells

**FIGURE 5:**
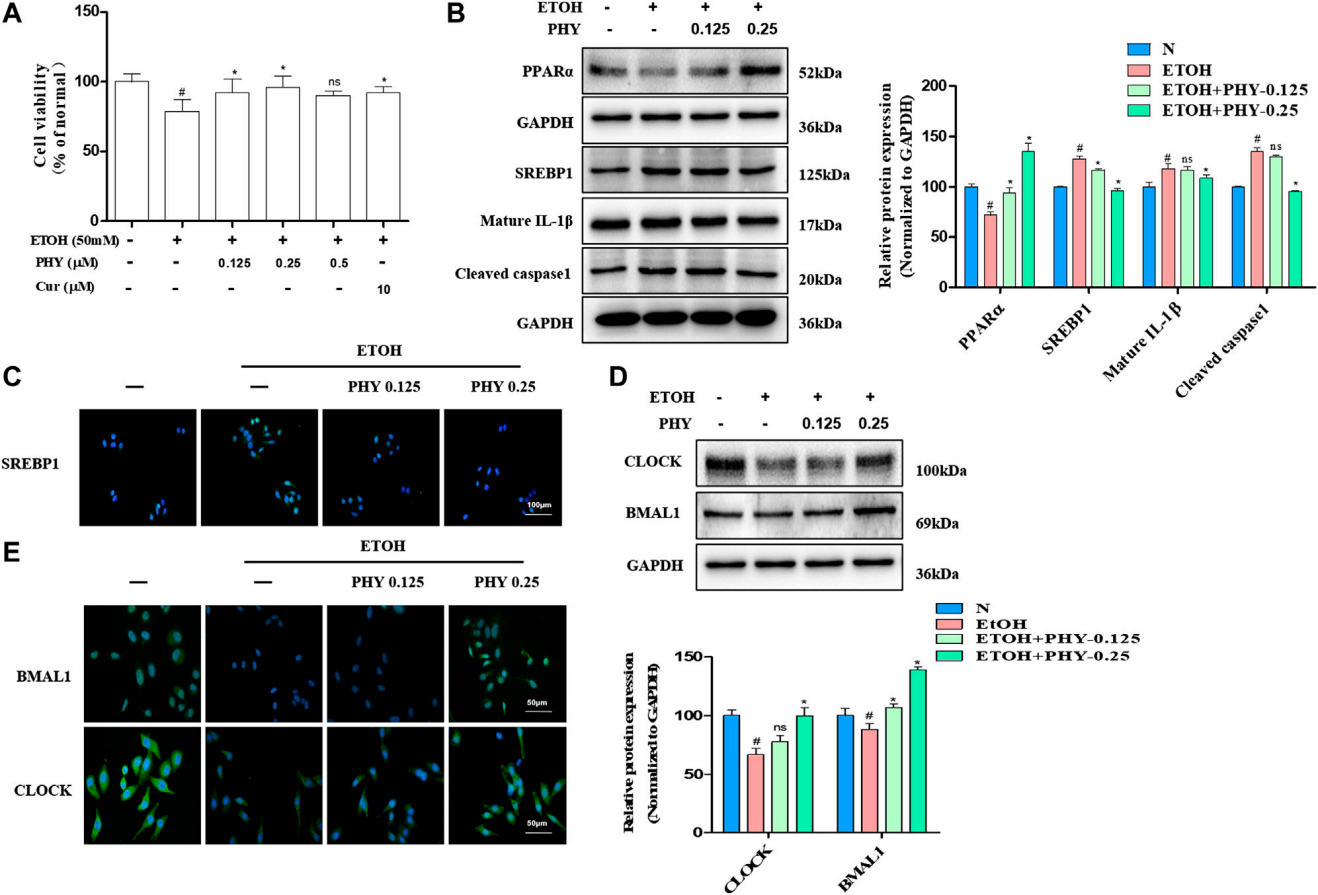
Physcion improves increased inflammasome activation and ameliorates lipid accumulation in HepG2 cells. **(A)** Cell viability was determined by MTT assay. **(B)** The protein expression of PPARα, SREBP1, IL1β, and caspase1. **(C)** Immunofluorescence staining of SREBP1. **(D)** The protein expression of BMAL1 and CLOCK. **(E)** Immunofluorescence staining of BMAL1 and CLOCK. Values significantly different from the control are indicated by hash signs (^#^ *p* < 0.05). Values significantly different from the ethanol are indicated by asterisks (*p < 0.05).

Physcion dose-dependently restored ethanol-inhibited cell survival in HepG2 cells (Figure 5A). The lipid accumulation induced by ethanol is modulated by PPARα-mediated fatty acid oxidation and SREBP1-mediated lipogenesis. As expected, SREBP1 expression levels were increased after ethanol exposure, and ethanol inhibited PPARα protein expression in HepG2 cells (Figures 5B,C). However, the inhibitory of SREBP1 was restored by Physcion. And ethanol suppressed PPARα levels were restored by Physcion. Excessive lipid accumulation results in inflammation with elevation of pro-inflammatory cytokines. Consequently, we measured how caspase-1 and IL1β expression is modulated by physcion in response to ethanol exposure. The IL1β expression and caspase-1 activity were markedly elevated in ethanol-induced HepG2 cells (Figure 5B). Nevertheless, the expression of IL1β and Caspase-1 were markedly reduced by Physcion administration.

To examine the association between circadian rhythm and hepatocyte steatosis, we use western blotting analysis test to examine the regulation effects of physcion on molecular circadian clock. As shown in Figure 5D, ethanol administration reduced the proteins expression of BMAL1 and CLOCK in HepG2 cells. However, physcion restored the proteins expression of circadian clock in HepG2 cells in comparison to ethanol-exposed cells. To further detect the distribution of BMAL1 and CLOCK in HepG2 cells, Immunofluorescence analysis was performed. The positive (in green) expressions of BMAL1 and CLOCK in EtOH group were decreased, while these were inhibited by Physcion treatment (Figure 5E).

### Effects of Physcion on Ethanol-Induced Circadian Dysregulation in HepG2 Cells

Previous studies have shown that several circadian clock genes exhibit a significant and clear 24-h rhythm (Bmal1, Cry1, Per2, Per1, and NR1D1) in HepG2 cells (Mazzoccoli et al., 2016). Furthermore, the disruption of lipid metabolism in HepG2 cells caused by free fatty acid can be ameliorated by cichoric acid (Guo et al., 2018). Studies have revealed that HepG2 cells contain class 4 alcohol dehydrogenase genes (ADH4) which can metabolises ethanol (Donohue et al., 2006; Pochareddy and Edenberg, 2012). Hence, HepG2 cells are suitable to be used as *in vitro* model system to test the influence of ethanol on the human body and to study *in vitro* lipid metabolism and the circadian rhythm.

**FIGURE 6:**
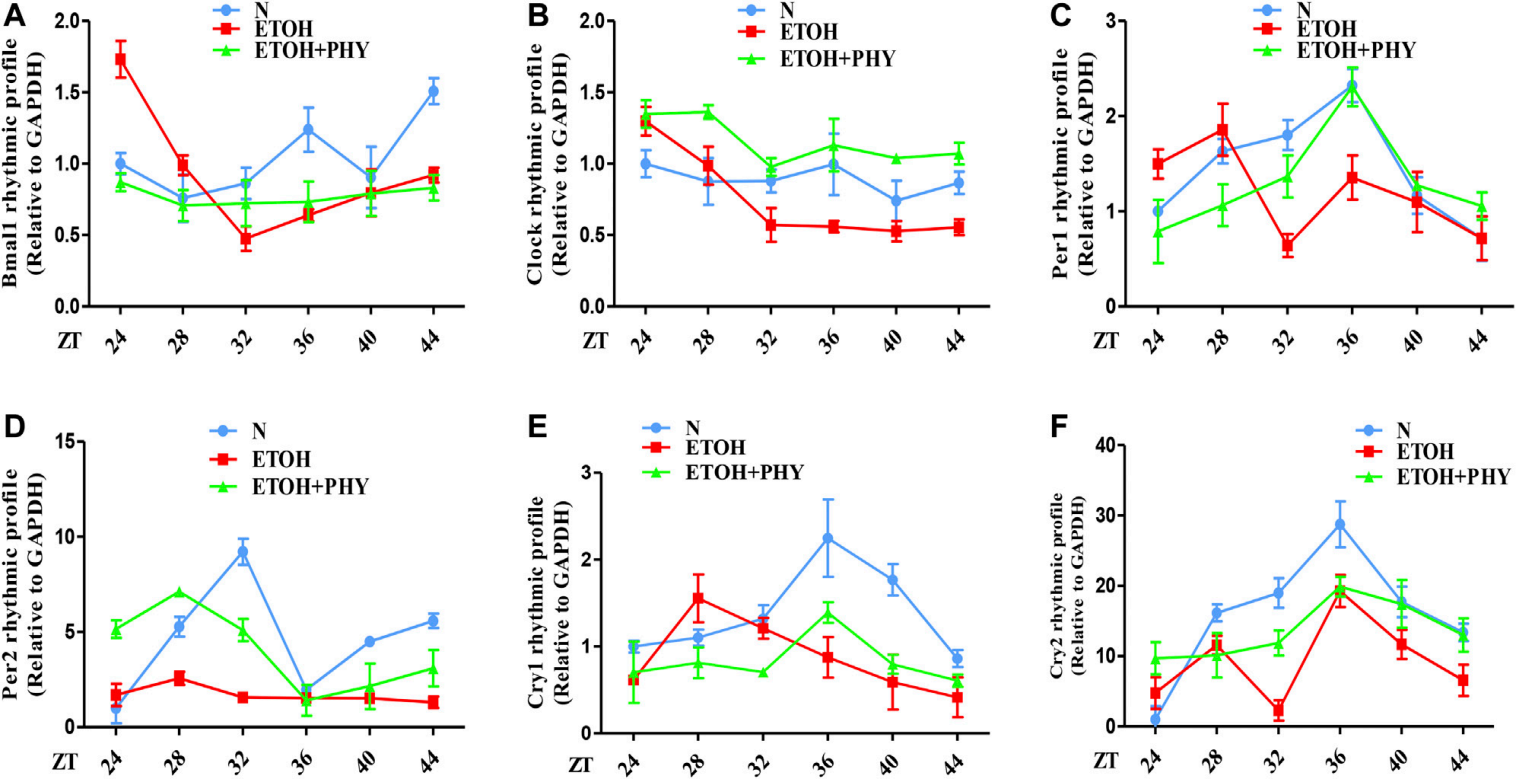
Effects of physcion on ethanol-induced circadian dysregulation in HepG2 cells. **(A–F)** The mRNA expression of Bmal1, Clock, Per1, Per2, Cry1, and Cry2 in HepG2 cells.

To reveal how Physcion exposure improve the mRNA oscillation of clock genes of HepG2 cells in ethanol, cells were cultured with ethanol and Physcion for 24 h after 2 h of serum shock. The strong rhythmic expressions of Clock, Bmal1, Per1, Per2, Cry1, and Cry2 were observed in the control group (Figures 6A–F). The mRNA levels of Clock and Bmal1 were similar to each other in cyclic oscillations, and both genes reached the highest levels at ZT36 to ZT44 (Figures 6A,B). The results showed that ethanol interfered with the daily expression of Clock, Bmal1, Per1, Per2, Cry1, and Cry2, and physcion effectively reversed this effect.

### Physcion Attenuates Intracellular Lipid Accumulation in a Bmal1-Dependent Way

**FIGURE 7:**
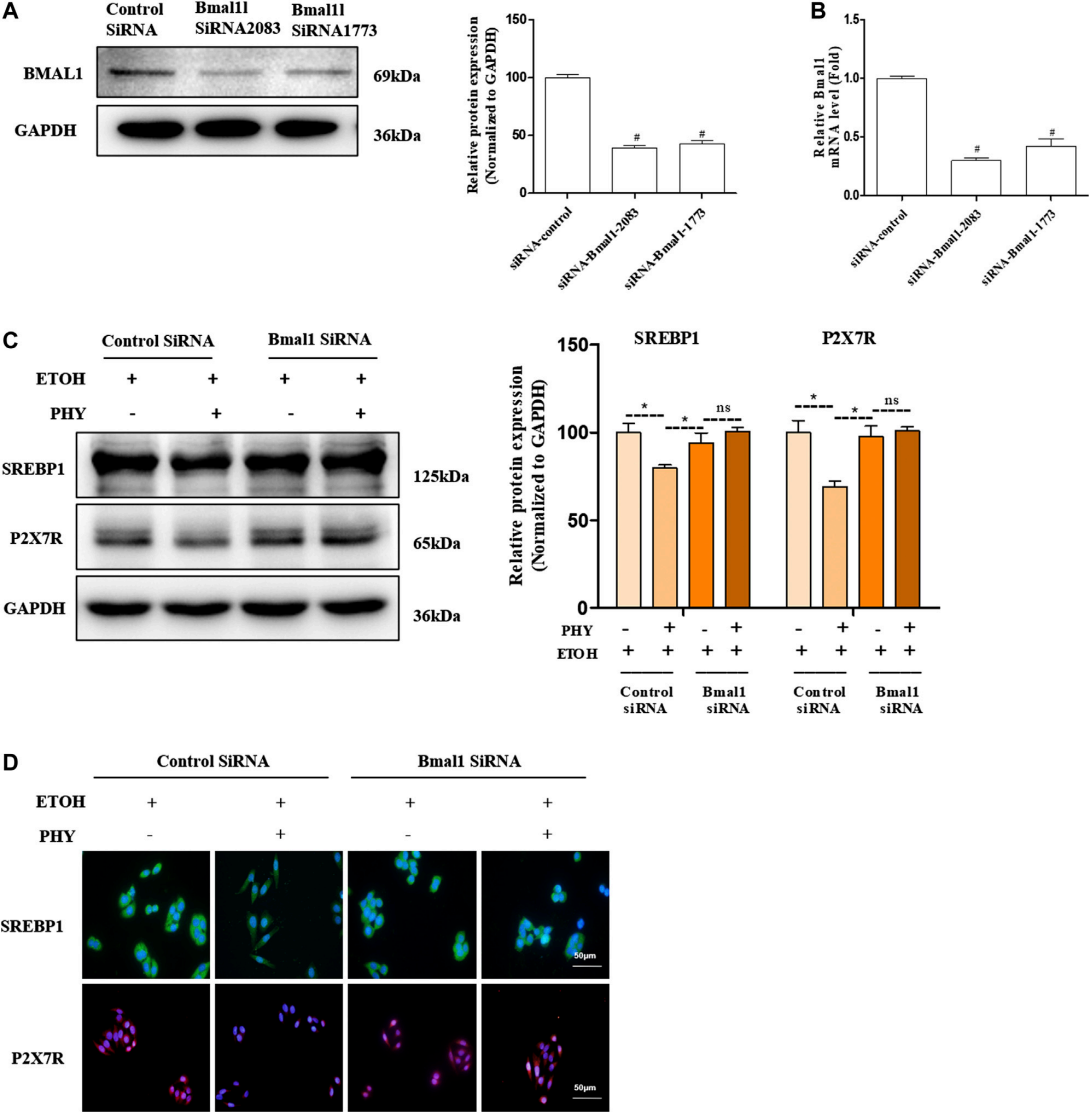
Physcion attenuates intracellular lipid accumulation in a Bmal1-dependent way. **(A)** The protein expression of BMAL1. **(B)** The mRNA expression of Bmal1. **(C)** The protein expression of SREBP1, and P2X7R. **(D)** Immunofluorescence staining of SREBP1 and P2X7R. Values significantly different from the control are indicated by hash signs (^#^ *p* < 0.05). Values significantly different from the ethanol are indicated by asterisks (*p < 0.05).

As presented in Figures 7A,B, siRNA against Bmal1 blocked BMAL1 protein and mRNA levels relative to control siRNA-transfected group. Physcion cotreatments significantly inhibited the increase of SREBP1and P2X7R induced by ethanol (Figure 7C). However, Physcion does not inhibit the expression of SREBP1 and P2X7R in the presence of silencing Bmal1. Consistently, si-Bmal1 suppressed the improvement of SREBP1 and P2X7R by physcion was shown by immunofluorescence analysis (Figure 7D).

**FIGURE 8:**
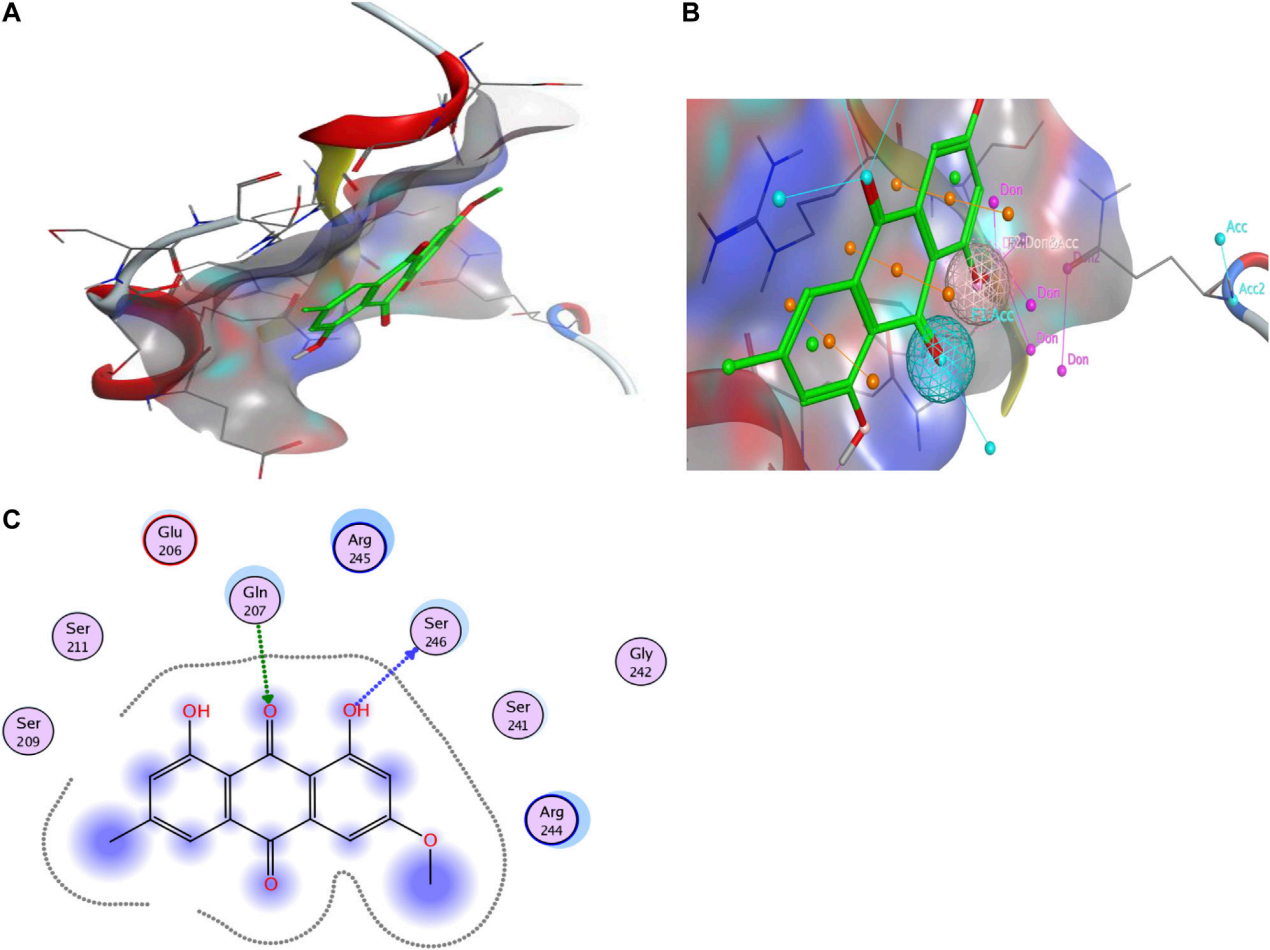
Computer analysis of **(A)** Bmal1 and Physcion, **(B)** molecular docking of Bmal1 and Physcion, and **(C)** interaction of active site and H-bond, 2-D interaction diagram.

The results of molecular docking also support these findings, indicating a high docking score and strong binding affinity for Bmal1 and Physcion. The lowest binding energy is (−4.6794 kcal mol^−1^) and it has a crucial interaction with the active pocket of Bmal1 (Figure 8). In addition, the two-dimensional image shows the formation of hydrogen bonds between Physcion and serine 246 and glutamine 207, with minimum distances of 3.08 and 2.96 Å. Tables 2, 3 provide other computer features.

**TABLE 2:** Descriptive properties of selected target protein for molecular docking.

Protein name	Uni-protID	PDB ID	Chain	Length	Gene	Classification
Bmal1	Q9WTL8	4F3L	B	632	Arntl	Transcriptional activator

**TABLE 3:** Bmal1 and Physcion hydrogen bonding.

Ligand group	Receptor	Interaction	Distance (Å)	E (kcal mol^−1^)
1-Dihydroxy	Ser 246 (B)	H-donor	3.08	−1.9
9-Dione	Gln 207 (B)	H-acceptor	2.96	−1.8

## Discussion

In this study, we show that Physcion resets the liver circadian clock and ameliorates hepatosteatosis and inflammation. Our findings on the liver protective effect induced by circadian alteration are congruent with the reported effect of Bmal1 on liver function (by Bmal1 knock out) (Zhang D. et al., 2018). Similarly, our results, i.e., that Physcion induced rhythmic expression of clock proteins such as Bmal1 and PER2, are compatible with the previous reports that core circadian genes are tightly linked to metabolic and inflammatory pathways.

The circadian rhythm is driven by diverse processes including fatty acid oxidation and synthesis, glucose metabolism, mitochondrial function and tricarboxylic acid cycle (Panda et al., 2002; Eckel-Mahan et al., 2012). Changes in these processes precipitate diurnal variations in various metabolites. Lipid metabolism involves many key transcriptional regulators, which are controlled by the biological clock and exist in the liver and adipose tissue. Lipid metabolism disorders, obesity and metabolic diseases are the consequences of circadian rhythm disorders. The research showed that a high-fat diet obviously inhibited the hepatic circadian clock (Kohsaka et al., 2007).

Hepatic steatosis precedes the development of alcoholic fatty liver (Zhang Y. et al., 2018). AMPK is a key multi-protein complex that controls liver lipid homeostasis (Gnocchi et al., 2015). AMPK is directly related to the regulation of the circadian clock (Gnocchi et al., 2015). To investigate whether and how Physcion can improve alcoholic fatty liver degeneration *in vivo*, mouse models of acute alcoholic steatosis were established by triple intragastric administration of ethanol to the stomach. Acute alcohol therapy can disrupt the molecular clock of the liver and reduce the expression of AMPK and Bmal1. Ethanol binding inhibits Bmal1, which leads to inhibition of AMPK phosphorylation and enhanced SREBP1 transcriptional activity, resulting in increased lipid synthesis and reduced fatty acid oxidation in the liver. Our data show that Physcion eliminates SREBP1 in acute ethanol-induced hepatic steatosis by modulating Bmal1 and AMPK. Activated AMPK is directly phosphorylated to LKB1 kinase associated Cry1 and is involved in the regulation of lipid biosynthesis. As expected, Physcion increased AMPK phosphorylation and inhibited SREBP1 expression. These results indicate that Physcion alleviates acute alcoholic fatty liver by promoting fatty acid oxidation and regulates Bmal1 to inhibit lipogenesis. It has been reported that PPARα dysfunction is related to ethanol-induced steatohepatitis (Li et al., 2019). It is interesting that we also observed Physcion reversed alcohol mediated PPARα reduction. Further experiments deserve to test whether Physcion has second impact on PPARα.

As peripheral circadian clocks have been reported to be involved in phlogistic pathways, a disrupted circadian rhythm contributes to the onset of various inflammation-related disease states (Jahanban-Esfahlan et al., 2018). Physcion has been reported to alleviate inflammation, to possess anti-cancer, laxative and anti-microbial activities. In this study, we found that Physcion targets Bmal1 to regulate the adipocytic pathway in hepatocytes and to limit the production of the pro-inflammatory cytokine IL1β. Specifically, we uncovered that Physcion improves circadian rhythm misalignment and relative slight alcohol-induced oscillations of clock genes in HepG2 cells. In addition, we found that Physcion can activate AMPK and Bmal1, inhibit the expression of SREBP1, eliminate lipid accumulation and inhibit the release of the pro-inflammatory factor IL1β. Moreover, our results show that a decrease in Bmal1 activity leads to a disruption of lipid metabolism and an abnormal production of IL1β, thereby explaining why HepG2 cells lacking Bmal1 have a higher pro-inflammatory response to alcohol.

**FIGURE 9:**
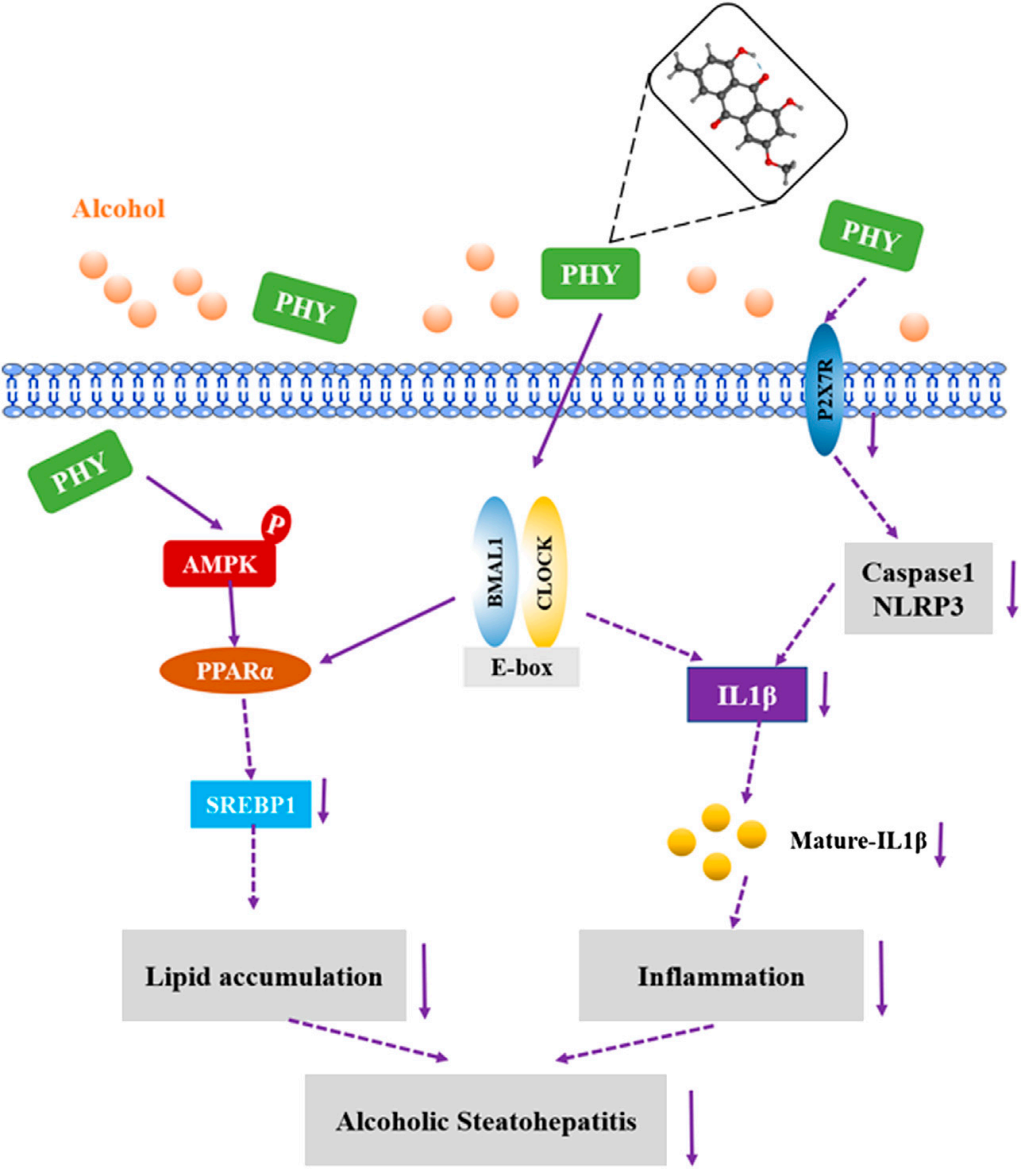
Possible mechanism(s) of suppression of alcoholic hepatosteatosis by physcion. Activation of AMPK-PPARα signaling and inhibition of P2X7R-IL-lβ inflammasome by physcion might be mediated by circadian clock pathway.

Based on the observations of our *in vitro* study, Physcion can directly alleviate the inhibition of the circadian pathway induced by alcohol in the HepG2 cells. Alcohol treatment can disrupt the central clock and lead to arrhythmia. To understand whether Physcion indirectly impacts the peripheral hepatic clock by the central circadian clock, further study is required. Physcion can protect the body from various sorts of damage, however, little is known regarding the role of Physcion in ethanol-induced damage in hepatic cells. Resveratrol has been found to alleviate rhythmic amplitude induced by high fat levels (Sun et al., 2015). Cichoric acid suppresses lipid metabolic disorders by modulating BMAL1 in HepG2 cells (Guo et al., 2018). In this study, Physcion was found to prevent alcoholic hepatosteatosis through regulating the circadian clock protein Bmal1 (Figure 9). To the best of our knowledge, this is the first report to provide evidence that Physcion can reprogrammed the biological clock to improve alcohol-induced liver injury.

Taken together, our study shows that Physcion, a natural circadian clock modulator, regulates fat synthesis and inflammation. This study provides clues to the role and molecular mechanism of Physcion in preventing ALD, and provides the possibility for the development of *Rheum emodi* as a functional food to prevent ALD.

## Data Availability Statement

The raw data supporting the conclusions of this article will be made available by the authors, without undue reservation.

## Author Contributions

YY, YZ, and AZ are the primary investigators in this study. AZ and QD participated in part of animal experiments. TZ and SL participated in part of culture cell experiments. MW, HX, and JM participated in part of data analysis. YZ designed this study.

## Funding

This work was supported by grants from the Shenzhen Science Technology and Innovation Commission (JCYJ20180305163454959 and JCYJ20190808155218940), Natural Science Foundation of Guangdong Province (2016A030310037) and Guangdong Basic and Applied Basic Research Foundation (2019A1515110006).

## Conflict of Interest

The authors declare that the research was conducted in the absence of any commercial or financial relationships that could be construed as a potential conflict of interest.
